# p300: expanding beyond acetylation to mastermind lactylation-dependent tumorigenesis

**DOI:** 10.3389/fcell.2026.1811421

**Published:** 2026-05-13

**Authors:** Su-Ting Jiang, Xiao He, You-Wei Wang, Wan-Song Chen, Peng Guan

**Affiliations:** 1 School of Medical Technology, Chongqing Three Gorges Medical College, Chongqing, China; 2 Chongqing Key Laboratory of Development and Utilization of Genuine Medicinal Materials in Three Gorges Reservoir Area, Chongqing Three Gorges Medical College, Chongqing, China; 3 Chongqing Engineering Research Center of Antitumor Natural Drugs, Chongqing Three Gorges Medical College, Chongqing, China; 4 School of Basic Medical Sciences, Chongqing Three Gorges Medical College, Chongqing, China

**Keywords:** cancer, epigenetics, histone modification, lactylation, p300

## Abstract

The transcription coactivator p300 is canonically recognized as a histone acetyltransferase that orchestrates chromatin remodeling and gene expression. However, recent breakthroughs have expanded the catalytic landscape of p300 beyond acetylation to include lactylation, a metabolic stress induced modification driven by lactate accumulation. As a principal lactyl-transferase, p300 acts as a sensor of the Warburg effect, directly translating cellular metabolic status into epigenetic regulation. This catalytic duality extends beyond histones to non-histone substrates, profoundly impacting tumor progression, immune evasion, and metabolic adaptation. This review synthesizes the established roles of p300 in acetylation while critically examining its emerging identity as a writer of protein lactylation. By highlighting the competitive interplay between acetyl-CoA and lactyl-CoA usage, we position p300 as a central integrator that couples metabolic signaling with transcriptional reprogramming in cancer and other pathological states. Furthermore, this review bridges the gap between mechanistic discovery and clinical translation by evaluating the therapeutic potential of targeting p300 lactyl-transferase activity. We highlight the emerging clinical relevance of p300 inhibitors, such as CCS1477 (in Phase I/II trials) and the preclinical agent A-485, discussing how these compounds, originally designed for acetylation or bromodomain inhibition, may be repurposed to dismantle lactylation-driven oncogenic networks and overcome metabolic immune evasion in refractory tumors.

## 1 Introduction

Accumulating evidence indicates that epigenetic dysregulation represents a critical driving force in tumor initiation and progression (Mir et al., 2025). With the rapid advancement of high-throughput omics technologies, an increasing number of key proteins involved in epigenetic regulation have been identified. These proteins include DNA methyl-transferases (DNMTs), lysine demethylases (KDMs), and histone acetyltransferases (HATs) (Ocker et al., 2019). Among these, p300, a canonical member of the HAT family, is frequently discussed together with its homolog CREB-binding protein (CBP) as the p300/CBP complex (Zhu et al., 2023). The core function of this complex lies in catalyzing lysine acetylation of both histone and non-histone proteins, thereby promoting chromatin relaxation and acting as a transcriptional co-activator to enhance target gene expression (Wu et al., 2025b). Under physiological conditions, p300-mediated acetylation participates in multiple essential cellular processes, including cell cycle regulation, differentiation, and stress responses. However, in the context of cancer, aberrant enhancement or imbalance of p300 activity often leads to inappropriate oncogene activation or impairment of tumor suppressive pathways (Chava and Wajapeyee, 2025; Chen et al., 2022). For instance, by modulating the acetylation status of key transcription factors such as p53, p300 can promote tumor cell proliferation, resistance to apoptosis, and therapeutic tolerance, ultimately driving tumor initiation and progression. These characteristics position p300 as a pivotal entry point for current studies on cancer epigenetics and targeted therapeutic intervention.

Notably, the tumor microenvironment is not static; rather, its metabolic state undergoes profound alterations during tumor progression and immune activation. Among these changes, enhanced aerobic glycolysis accompanied by excessive lactate accumulation represents a hallmark feature of metabolic reprogramming in cancer (Alzial et al., 2021). Unlike normal cells, which primarily rely on oxidative phosphorylation for ATP pro-duction, tumor cells preferentially utilize glycolysis to generate energy even under oxygen-sufficient conditions—a phenomenon known as the Warburg effect (Nishida and Andoh, 2025). Although this metabolic strategy yields relatively limited ATP, it results in substantial accumulation of lactate both intracellularly and extracellularly. Traditionally, lactate has been regarded as a metabolic waste product or an auxiliary energy substrate. However, emerging evidence has revealed that lactate itself can function as a signaling molecule, deeply involved in transcriptional regulation and epigenetic remodeling (Cheng et al., 2020). This paradigm shift raises a fundamental scientific question: within a lactate-rich tumor microenvironment, does p300 merely retain its canonical role as an acetyltransferase, or does its functional repertoire expand to mediate novel, metabolism-dependent epigenetic regulatory modes?

The answer to this question began to emerge with a landmark study published in 2019. The study first demonstrated that lactate can serve as a precursor for post-translational modification by forming lactyl-CoA, thereby enabling lysine lactylation of proteins, with p300 identified as the key writer enzyme responsible for this modification (Zhang et al., 2019). This discovery broadened the biological significance of lactate and fundamentally reshaped the conventional understanding of p300 function. p300 is no longer viewed solely as an acetyltransferase; instead, it is recognized as a dual-functional epigenetic regulator capable of sensing cellular metabolic states and dynamically switching between acetylation and lactylation.

Beyond its fundamental biological significance, the identification of p300 as a lactyl-transferase reveals a tangible therapeutic vulnerability. The clinical urgency of this axis is underscored by the rapid development of small-molecule inhibitors targeting the p300 catalytic core and bromodomain. Notably, agents such as CCS1477 have already entered Phase I/II clinical trials for metastatic prostate cancer (Welti et al., 2021), while potent catalytic inhibitors like A-485 and C646 show promising efficacy in preclinical models of hematological and solid malignancies (Ji et al., 2022; Ono et al., 2021). Emerging evidence suggests that the therapeutic efficacy of these drugs may stem not only from suppressing acetylation but also from disrupting the lactylation-dependent metabolic memory of cancer cells (Jie et al., 2021; Di et al., 2019). Consequently, understanding the specific contribution of p300-mediated lactylation to tumor progression is a prerequisite for optimizing these next-generation epigenetic therapies.

Based on these advances, this review systematically summarizes the dual epigenetic roles of p300 in cancer. Following a revisitation of the classical p300-mediated acetylation networks and their functions in gene regulation and disease, attention is turned to the discovery of lactylation and its underlying molecular mechanisms. This section highlights how p300 acts as a metabolic–epigenetic bridge that directly links the Warburg effect to transcriptional control. The discussion culminates in an exploration of potential therapeutic strategies targeting the dual functional activities of p300 and an outline of key unresolved scientific questions in this rapidly evolving field. Through this comprehensive review, we aim to provide a clear conceptual framework for understanding the interplay between tumor metabolism and epigenetic regulation and to offer new insights for the development of precision-targeted therapeutic strategies.

## 2 The classical functions of p300: an epigenetic core acting as a transcriptional coactivator

p300 is a large, multi-domain protein whose functional complexity arises from its finely organized modular architecture. Structurally, p300 is composed of multiple do-mains with distinct and specialized functions, among which the histone acetyltransferase (HAT) domain serves as the catalytic core. This core is complemented by several regulatory and interaction modules, including the bromodomain (BD), RING domain, plant homeodomain (PHD), as well as the downstream ZZ and TAZ2 domains (Xu et al., 2023). These domains cooperatively fold in three-dimensional space to form a highly dynamic platform for substrate recognition and catalysis, enabling p300 not only to write acetylation marks but also to participate in the assembly of transcriptional regulatory complexes through specific domain-mediated interactions (Gou and Zhang, 2024). Within the classical epigenetic framework, p300 is primarily regarded as a lysine acetyltransferase that transfers acetyl groups from acetyl-CoA to lysine residues on histone and non-histone proteins. This acetylation neutralizes the positive charge of lysine residues, thereby weakening their interaction with the negatively charged DNA phosphate backbone and inducing chromatin relaxation. Such structural remodeling creates a permissive chromatin environment for the binding of transcription factors and RNA polymerase II, positioning p300 as an indispensable regulator of transcription initiation and enhancer activation (Liu R. et al., 2025).

**FIGURE 1:**
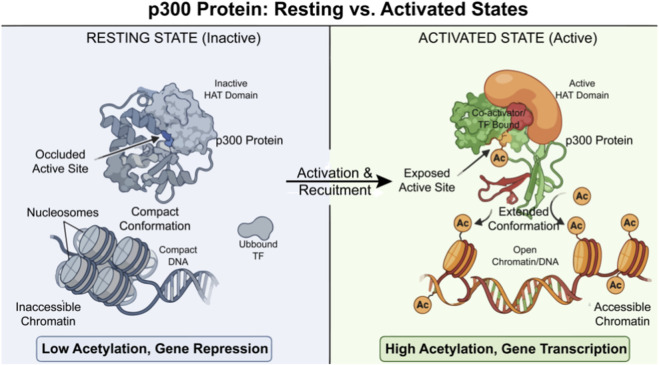
Conformational and functional states of p300: from an autoinhibited resting state to an active acetyltransferase. Left (resting/inactive): p300 adopts a compact, autoinhibited conformation in which the HAT active site is occluded, resulting in poor engagement with DNA-bound transcription factors (TFs) and other co-regulators. Nucleosomes remain compact, chromatin is in-accessible, histone acetylation is low, and gene transcription is repressed. Right (activated): p300 undergoes a conformational extension that relieves autoinhibition and exposes the HAT active site. Binding of transcription factors and/or co-activators stabilizes the active conformation and promotes recruitment. Active p300 acetylates nucleosomal histones (Ac), opening chromatin and increasing accessibility, thereby promoting gene transcription.

In the absence of external stimuli or transcription factor recruitment, p300 is generally maintained in a low-activity state where its HAT domain is typically con-strained by an autoinhibitory conformation, thereby preventing non-specific and widespread acetylation (Becht et al., 2025). The presence of this autoinhibitory state allows p300 to remain highly responsive to upstream signaling cues and to be selectively activated only upon recruitment by specific transcription factors or changes in the chromatin landscape (Figure 1). This property is essential for enabling p300 to participate in diverse biological processes under both physiological and pathological conditions without triggering catastrophic transcriptional dysregulation. In the oncogenic context, the canonical acetylation functions of p300 are frequently reprogrammed. On one hand, aberrantly elevated p300 activity can promote tumor initiation and progression by driving onco-gene transcription through excessive histone acetylation. On the other hand, under certain circumstances, p300 may exert tumor-suppressive effects by activating tumor suppressor genes or inducing apoptosis. It is precisely this context-dependent mode of acetylation regulation that renders p300 one of the most controversial yet therapeutically promising nodes in cancer epigenetic research.

In the following sections, we will first focus on p300-mediated histone acetylation and systematically summarize its roles in tumor initiation, progression, and immune regulation. We will then extend the discussion to non-histone acetylation, elucidating how p300 directly reshapes the activity and stability of key functional proteins to orchestrate multiple dimensions of malignant phenotypes.

### 2.1 p300-mediated histone acetylation: chromatin plasticity and transcriptional reprogramming in cancer

Histone acetylation represents one of the most classical and extensively studied functions of p300 and constitutes the molecular basis for its role as a transcriptional co-activator. The functions of p300 as more than just a background chromatin modifier; it is a tightly regulated enzyme that executes precise acetylation at specific genomic loci and time points. This spatiotemporal control drives the reorganization of transcriptional programs. Among the numerous histone acetylation sites, p300 predominantly catalyzes acetylation of lysine 18 and lysine 27 on histone H3 (H3K18ac and H3K27ac) (Kyrchanova et al., 2023). These modifications are widely recognized as hallmark epigenetic sig-natures of active promoters and enhancers, with elevated levels closely correlated with enhanced transcriptional output (Xu et al., 2025). Under normal physiological conditions, p300 is precisely recruited to genomic loci associated with development, differentiation, or stress responses, where it transiently induces H3K18ac and H3K27ac, enabling reversible and tightly controlled transcriptional regulation.

In the tumor context, however, this finely tuned regulatory mechanism is frequently disrupted. Aberrant activation or sustained recruitment of p300 leads to excessive accumulation of H3K18ac and H3K27ac at specific genomic regions, triggering widespread transcriptional reprogramming that ultimately drives tumor initiation and malignant progression (Yu et al., 2023). For example, in angioimmunoblastic T-cell lymphoma, p300-mediated hyperacetylation of H3K27 results in persistent IL-2 overexpression, thereby promoting uncontrolled tumor cell proliferation (Vallois et al., 2025). In colorectal cancer, IFN-γ–activated phosphorylated STAT1 (pSTAT1) recruits p300 to the GPR109A promoter, inducing excessive H3K18 acetylation and suppressing GPR109A expression, which in turn facilitates tumorigenesis (Bardhan et al., 2015). Similarly, in prostate cancer, p300-driven acetylation of H2B and H3K27 cooperatively sustains oncogenic transcriptional pro-grams, markedly enhancing tumor invasiveness (Luo et al., 2025).

Beyond direct regulation of intrinsic transcriptional programs in tumor cells, p300-mediated histone acetylation also plays a critical role in shaping the tumor immune microenvironment. Recent studies have demonstrated that tumor-derived exosomes, as key mediators of intercellular communication, can reprogram the transcriptional states of recipient cells through p300-dependent epigenetic mechanisms. For instance, exosomal LINC01232 secreted by tumor-associated macrophages directly binds the transcription factor E2F2 and promotes its nuclear accumulation, cooperatively enhancing transcription of NBR1. NBR1 facilitates autophagic degradation of MHC-I proteins, thereby reducing surface MHC-I expression on tumor cells and enabling immune evasion from CD8^+^ T cell-mediated cytotoxicity (Li et al., 2023). In colorectal cancer, exosomal LINC01232 also interacts with p300 to elevate H3K27ac levels at the ARNTL2 promoter, thereby suppressing ferroptosis and promoting tumor cell survival (Zhuang et al., 2024).

p300-mediated H3K27ac has likewise been shown to directly regulate the expression of immune checkpoint molecules. Enrichment of H3K27ac at the PD-L1 promoter enhances PD-L1 transcription, leading to attenuation of antitumor immune responses and posing a significant obstacle to immunotherapy in multiple cancers, including melanoma (Wang et al., 2024). In addition, H3K27ac can promote perineural invasion through the E2F1/GDNF axis, providing both structural and signaling support for pancreatic cancer metastasis (Gu et al., 2025).

A critical nuance is that p300-mediated histone acetylation does not universally promote tumorigenesis. Under specific tumor types and signaling contexts, p300 can exert tumor-suppressive functions. For example, in gastric cancer, p300 forms a complex with the transcription factor Sp1, inducing high levels of histone H3 and H4 acetylation at promoter regions and thereby activating transcription of the tumor sup-pressor gene RASSF2A, which triggers apoptosis in cancer cells (Liu et al., 2010). Similarly, in breast cancer, enhanced p300 activity increases levels of H3K9ac, H3K14ac, and H3K27ac, leading to apoptosis induction and suppression of tumor formation (Zamperla et al., 2024).

Beyond cancer, p300-mediated H3K18ac and H3K27ac are broadly involved in a wide range of physiological and pathological processes, including stem cell differentiation, metabolic inflammation, and immune response regulation. For instance, H3K18ac and H3K27ac play essential roles in osteogenic differentiation of human adult mesenchymal stem cells and pluripotent stem cell differentiation, respectively (Zeng et al., 2025; An et al., 2025). Moreover, p300-induced H3K18 acetylation promotes IL-6 production in response to lipopolysaccharide stimulation, thereby exacerbating metabolic inflammation and in-creasing obesity risk (Kochumon et al., 2022).

Collectively, p300-mediated histone acetylation is not a simple switch but rather a sophisticated, context-dependent regulatory mechanism defined by cell lineage, signaling cues, and the target gene repertoire. This functional plasticity positions p300 as a critical node in tumorigenesis, immune regulation, and metabolic remodeling. Importantly, such catalytic flexibility also lays the mechanistic basis for the broad role of p300 in mediating non-histone acetylation and novel acylations, particularly lactylation.

### 2.2 p300-mediated non-histone acetylation: an epigenetic extension that directly reprograms protein function

Unlike histone acetylation, which indirectly regulates gene transcription through modulation of chromatin structure, p300-mediated non-histone acetylation confers a more direct, rapid, and highly specific regulatory modality. By acetylating lysine residues on key functional proteins, p300 can directly alter protein conformation, stability, subcellular localization, and affinity for interacting partners without relying on chromatin remodeling (Lee et al., 2023). Through this mechanism, p300 fine-tunes cellular signaling transduction and cell fate decisions, thereby expanding its functional scope from a transcriptional regulator to a protein function reprogramming factor. From the perspective of substrate recognition, p300-mediated non-histone acetylation does not occur randomly but instead follows a highly selective molecular logic. In contrast to its regional scanning behavior on chromatin, p300 typically recognizes specific sequence motifs or structural features within non-histone substrates through its multiple protein–protein interaction domains, enabling precise targeting. This strategic shift from regional localization to molecular recognition is central to understanding the biological significance of p300-mediated non-histone acetylation.

p53 represents the most classical and extensively studied model of p300-mediated non-histone acetylation (Feng and Meng, 2022). As a central tumor suppressor, p53 rapidly stabilizes and ac-cumulates in the nucleus in response to cellular stress such as DNA damage or onco-gene activation (Kobet et al., 2000; Grossman, 2001). During this process, upstream kinases ATM and ATR phosphorylate multiple serine residues at the N-terminus of p53, thereby enhancing its affinity for p300 (Wang Y-H. et al., 2022b). Phosphorylated p53 engages p300 with high affinity through interactions between its N-terminal transactivation domain and the KIX and TAZ1/CH1 domains of p300, recruiting p300 to the promoters of p53 target genes such as p21 and PUMA (Okuda and Nishimura, 2015). Subsequently, p300 acetylates key lysine residues (K373 and K382) within the intrinsically disordered C-terminal region of p53 via its HAT domain (Kamada et al., 2020). This modification relieves autoinhibitory constraints on the p53 DNA-binding domain, rendering its conformation more favorable for stable association with target gene promoters. Moreover, the acetylation of p53 directly competes with its ubiquitination. p300-mediated acetylation occupies critical lysine residues required for MDM2-dependent ubiquitination, thereby markedly suppressing p53 proteasomal degradation and extending its protein half-life (Gong et al., 2024; Lindström et al., 2022). In addition, acetylated p53 can be recognized by bromodomain-containing proteins, further amplifying transcription-al activation signals. Ultimately, through the coordinated actions of non-histone acetylation–enhanced transcription factor activity and histone acetylation–optimized chromatin accessibility, p300 achieves efficient and highly specific activation of p53 target genes, leading to cell fate decisions such as cell cycle arrest, DNA repair, or apoptosis (San José-Enériz et al., 2019). Collectively, these acetylation-mediated mechanisms constitute a crucial barrier against disease, with their dysregulation being a hallmark of tumorigenesis. For example, in glioblastoma, p53 acetylation has been proposed as a favorable prognostic molecular marker. Conversely, within the competitive Smad1-p300-p53 complex, the oncogenic protein Smad1 preferentially acquires p300-mediated acetylation at K373, thereby suppressing p53 acetylation and promoting tumor growth and chemo-resistance (Gong et al., 2024). In pancreatic cancer, the acetylation of p53 is suppressed by the SET/TAF-Iβ–p300 interaction, thereby enhancing tumor cell survival (Di Crosta et al., 2025). This phenomenon further underscores the critical role of substrate competition for p300 in tumorigenesis.

In addition to p53, p300 acetylates a broad range of non-histone proteins to directly influence key processes such as tumor metabolism, transcriptional regulation, and immune evasion. For instance, acetylation of the core scaffold protein PDHX of the mitochondrial pyruvate dehydrogenase complex at K488 induces conformational changes that impair PDC assembly, thereby inhibiting the conversion of pyruvate to acetyl-CoA and ultimately promoting hepatocellular carcinoma progression (Jiang et al., 2024). In colorectal cancer, p300 acetylates the transcription factor FOXQ1 at K190 and cooperates with BRD4-recruited RNA polymerase II to form a transcriptional activation complex, markedly enhancing oncogene transcription and driving tumor proliferation and metastasis (Yang W-D. et al., 2025). p300-mediated non-histone acetylation can also contribute to tumorigenesis by modulating RNA processing and post-transcriptional regulation. For example, p300-dependent acetylation of PHF5A at K29 upregulates KDM3A expression through alternative splicing, thereby regulating stress responses and promoting colorectal cancer progression (Wang et al., 2019). In breast cancer cells, p300 acetylates Api5 at K251 to maintain its anti-apoptotic activity, enabling sustained cancer cell survival under nutrient deprivation or stress conditions (Sharma and Lahiri, 2021).

In addition to promoting intrinsic malignant phenotypes, p300-mediated non-histone acetylation plays a critical role in tumor immune evasion. Mye-loid-derived suppressor cells (MDSCs), a major immunosuppressive population within the tumor microenvironment, rely in part on p300-mediated acetylation of C/EBPβ for their suppressive function. This modification enhances C/EBPβ-driven transcriptional activation of the Arg1 promoter, thereby reinforcing MDSC-mediated suppression of T cell responses (Wang W. et al., 2022). In prostate cancer, p300-mediated acetylation of JMJD1A at K421 has also been shown to enhance therapeutic resistance (Xu et al., 2020). While p300 often acts as a master regulator, its own activity can be subject to upstream modulation, adding an-other layer of complexity to its role in cancer progression. Recent study has shown that the RNA acetyltransferase NAT10 can enhance p300 acetylation levels by modifying chromatin-associated tRNAs, a mechanism that promotes tumor cell lung metastasis (Amin et al., 2025). This example illustrates that the impact of p300 on tumor immune evasion is not always direct but can be mediated through such regulatory cascades.

While p300-mediated non-histone acetylation is a key oncogenic driver, it is important to note that its effects are not invariably tumor-promoting. In certain contexts, this modification can exert tumor-suppressive effects. For example, p300 acetylates the lysine metabolism enzyme glutaryl-CoA dehydrogenase (GCDH) at K438, leading to elevated intracellular reactive oxygen species levels and inhibition of oxidative phosphorylation. This metabolic disruption subsequently triggers ATR/Chk1-mediated impairment of DNA damage repair and activation of autophagy, ultimately suppressing hepatocellular carcinoma cell proliferation (Tian et al., 2025).

Collectively, these findings highlight p300-mediated non-histone acetylation as a rapid-response regulatory layer distinct from chromatin control, enabling cells to swiftly modulate protein function without *de novo* transcription. To synthesize the regulatory events of p300 in both histone and non-histone acetylation discussed in this section, we have compiled a summary table (Table 1) outlining its key physiological functions, serving as a quick-reference guide. However, this enzymatic versatility raises a pivotal question: In the lactate-rich tumor microenvironment, where the promiscuous catalytic pocket of p300 is prone to competitive occupation by lactyl-CoA, does p300 undergo an identity crisis? Specifically, does this metabolic saturation fundamentally hijack p300, forcing it to shift from a canonical acetyltransferase to a driver of pathogenic non-canonical modifications?

**TABLE 1:** Functional Landscape of p300-Mediated Acetylation in Cancer. This table summarizes specific lysine acetylation sites catalyzed by p300 on histone and non-histone substrates, their direct molecular or pathway-level functions, and the associated cancer types as reported in the cited literature. The Function column describes the primary biological consequence of the acetylation event.

Substrate (histone/Non-histone)	Acetylation site	Function	Cancer type	Ref.
H3	K18ac, K27ac	Activates enhancers and oncogene transcription	Cancers	Xu et al. (2025), Qian et al. (2023)
		Upregulates glycolysis-related enzymes	HCC	Cai et al. (2020)
		Inhibits IL-2 secretion	TFH cell lymphoma	Vallois et al. (2025)
		Modulates the Notch signaling pathway and EMT	BC	Sarkar et al. (2024)
H3	K18ac	Suppresses GPR109A expression	CRC	Bardhan et al. (2015)
H2B	ac	Sustains oncogenic transcription	PCa	Luo et al. (2025)
H3	K27ac			
H3	K27ac	Promotes Wnt/β-catenin pathway (via CREPT)	CRC	Zhang et al. (2018)
H4	ac			
H3	K27ac	Activates oncogenic transcription via TFAP2β in CRC	Neuroblastoma	Durbin et al. (2021)
		Represses STAT1 pathway and promotes migration/invasion via SOCS5	Osteosarcoma	Zhang et al. (2021)
		Mediates evasion from CD8^+^ T cell cytotoxicity	Glioma	Li et al. (2023)
		Suppresses ferroptosis	CRC	Li et al. (2023), Zhuang et al. (2024)
		Alters chromatin structure	Melanoma	Emmons et al. (2023)
		Activates the PI3K-AKT-mTOR axis	CRC	Liu (2023)
		Attenuates antitumor immune responses via PD-L1	Melanoma	Wang et al. (2024)
		Promotes perineural invasion via the E2F1/GDNF axis	PACA	Gu et al. (2025)
		Upregulates PYCR1 expression	GC	Xiao et al. (2022)
		Enhances 5-FU resistance via CD109 and JNK/MAPK signaling pathway	GC	Zhou et al. (2023)
		Promotes osteo/chondrogenic differentiation	Polyploid Giant Cancer	Yang et al. (2024b)
H3	K9ac, K14ac, K27ac	Induces cancer cell apoptosis	BC	Zamperla et al. (2024)
H3	K14ac	Promotes cell proliferation	GC	Wang et al. (2017)
H3	K9ac	Upregulates HMGA2 expression, promotes tumor metastasis	HCC	Liang et al. (2020)
p53	K373ac, K382ac	Induces cell cycle arrest, DNA repair, and apoptosis	Cancers	Kamada et al. (2020)
	K381ac, K382ac	Activates PUMA transcription and kills tumors	Cancers (GC, CRC)	Wang et al. (2023b)
Smad1	K373ac	Promotes tumor growth and chemoresistance	Glioblastoma	Gong et al. (2024)
PDHX	K488ac	Disrupts PDC assembly, inhibits acetyl-CoA production	HCC	Jiang et al. (2024)
FOXQ1	K190ac	Forms transcriptional activation complex with BRD4/Pol II	CRC	Yang W-D. et al. (2025)
PHF5A	K29ac	Modulates RNA processing and post-transcriptional regulation	CRC	Wang et al. (2019)
Api5	K251ac	Anti-apoptotic, inhibits cell death	BC	Sharma and Lahiri (2021)
C/EBPβ	—	Suppresses T cell responses	Cancers	Wang W. et al. (2022)
JMJD1A	K421ac	Enhances therapeutic resistance	PCa	Xu et al. (2020)
GCDH	K438ac	Induces metabolic stress and autophagy, suppressing tumor growth	HCC	Tian et al. (2025)

## 3 Advances in lactylation research: a metabolism-driven paradigm shift in epigenetic regulation

**FIGURE 2:**
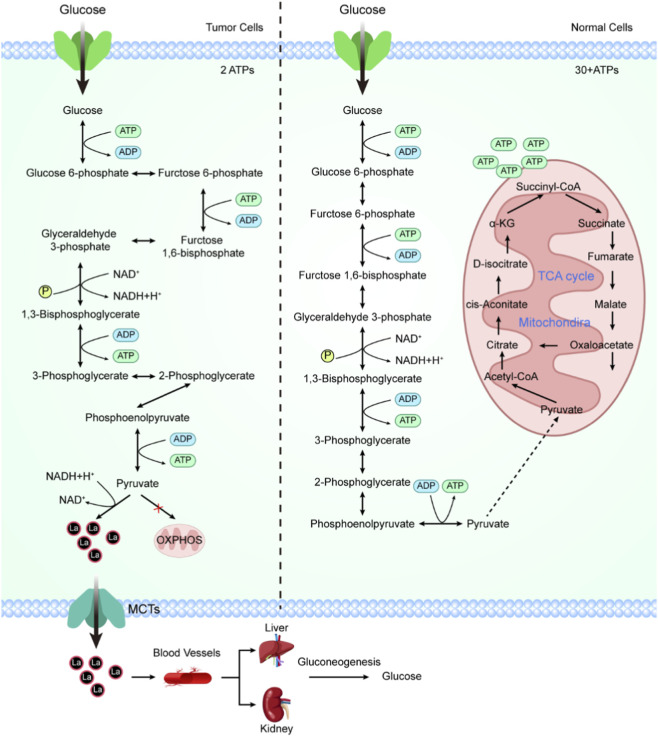
Comparison of Glucose Metabolism Pathways in Tumor and Normal Cells. Overview of glucose metabolism in tumor cells versus normal cells. The schematic illustrates the distinct metabolic pathways of glucose in tumor cells (left panel) and normal cells (right panel). In tumor cells, glucose is predominantly metabolized through aerobic glycolysis (Warburg effect), resulting in the production of lactate, even in the presence of oxygen. This process generates 2 ATP molecules per glucose molecule, with lactate exported via monocarboxylate transporters to the blood, where it can be recycled into glucose by the liver and kidneys through gluconeogenesis. In contrast, normal cells primarily metabolize glucose through OXPHOS within mitochondria, utilizing the TCA cycle to generate more than 30 ATP molecules per glucose molecule. Key intermediates and processes, including ATP generation, NAD+/NADH cycling, and lactate metabolism, are highlighted for both pathways.

For a long time, lactate was regarded as a metabolic byproduct of glycolysis, with its biological significance largely confined to the maintenance of energy homeostasis and the formation of an acidic microenvironment (Li et al., 2025b; Cuyàs et al., 2022). However, with the deepening understanding of tumor metabolism, this traditional view has gradually been overturned. Accumulating evidence indicates that lactate is not merely a passively accumulated metabolic waste product but rather an active metabolite with signaling functions that can directly participate in the regulation of cell fate (Yang R. et al., 2025). In particular, in tumor cells, the sustained high glycolytic flux driven by the Warburg effect provides a unique and stable metabolic foundation for lactate-mediated epigenetic regulation (Chetta et al., 2023). In multicellular organisms, efficient ATP production typically relies on mitochondrial oxidative phosphorylation. Tumor cells, however, reprogram their metabolism to overcome growth factor dependence and accommodate rapid proliferative demands, preferentially re-lying on glycolysis for energy generation even under oxygen-sufficient conditions. Although this metabolic strategy reduces the energetic yield per glucose molecule, it markedly accelerates metabolic flux and leads to substantial accumulation of lactate both intracellularly and extracellularly (Figure 2). It is within this lactate-rich context that lactate transitions from a metabolic endpoint to a signaling origin, thereby creating the necessary conditions for its involvement in epigenetic regulation.

The pioneering research by Professor Zhao’s team at the University of Chicago first systematically established lysine lactylation as a novel post-translational modification, a breakthrough that represents a pivotal turning point in metabolism and epigenetics (Zhang et al., 2019). This study demonstrated that lactate can be converted intracellularly into lactyl-coenzyme A through CoA-transfer reactions and subsequently serve as a donor for the covalent attachment of lactyl groups to lysine residues on proteins, thereby forming stable lactylation modifications. This discovery not only expanded the chemical repertoire of post-translational modifications but also mechanistically revealed how cellular metabolic states can be directly written into the protein modification landscape.

Similar to classical acetylation, lactylation is regulated by a comprehensive and dynamic enzymatic system comprising *writers*, *erasers* and *readers* (Zhao B. et al., 2025). These enzymes act in concert to maintain the homeostatic balance of histone and non-histone lactylation within cells, conferring high plasticity and reversibility to this modification (Tang et al., 2024). Within this framework, p300 acts as the critical metabolic transducer sensing the intracellular availability of lactyl-CoA and utilizing its promiscuous HAT domain to couple metabolic flux to epigenetic reprogramming (Zhang et al., 2019), thereby transforming lactate from a mere metabolite into a specific signaling code. However, the signaling output of p300 is strictly gated by a counterbalancing system of *erasers*, which serve to reset the p300-imposed lactylation landscape. Specific histone deacetylases (HDACs) and Sirtuins act as the necessary antagonists to ensure that p300-mediated signaling is both potent and reversible. For instance, HDAC1 and HDAC3 actively reverse p300-mediated lactylation (Carlos et al., 2022), with HDAC3 functioning as the dominant brake due to its superior catalytic efficiency (Zessin et al., 2022). In specific physiological contexts, SIRT1 and SIRT2 further refine this landscape by targeting non-histone substrates like HMGB1 or specific histone sites to restrain pathological progression (Yang et al., 2021; Zu et al., 2022).

The dynamic equilibrium between p300-mediated writing and eraser-mediated removal dictates the net density of the lactylation mark. Yet, for this metabolic code to elicit a biological response, it must be recognized by downstream effectors known as *readers*. Once the p300-driven signal successfully passes through the *eraser* gate, these marks remain functionally silent until decoded by specialized proteins capable of translating chemical modifications into transcriptional outputs. Pioneering studies first identified the chromatin remodeler Brg1 as the functional pioneer reader of histone lactylation. During cellular reprogramming, Brg1 specifically recognizes p300-catalyzed H3K18la at target promoters, actively remodeling chromatin to drive pluripotency and epithelial networks (Hu et al., 2024). While the discovery of Brg1 established the foundational paradigm of lactylation-dependent transcriptional control, understanding the broader structural basis for this recognition presents a unique stereochemical challenge. Historically, classical bromodomains, such as those found in BRD4, have served as the primary readers for lysine acetylation (Yuan et al., 2024). However, the lactyl group is both bulkier and more hydrophobic than its acetyl counterpart. This structural difference often creates steric clashes within the narrow binding pockets of conventional bromodomains. Recent structural biology breakthroughs have highlighted the YEATS domain family as highly optimized readers capable of circumventing this constraint. Characterized by an open-ended aromatic sandwich pocket, the YEATS domain readily accommodates bulkier acyl modifications with high affinity. Specifically, YEATS2 has emerged as a validated reader for p300-mediated lactylation (Pant et al., 2025), translating this metabolic-epigenetic mark into downstream oncogenic transcription.

**FIGURE 3:**
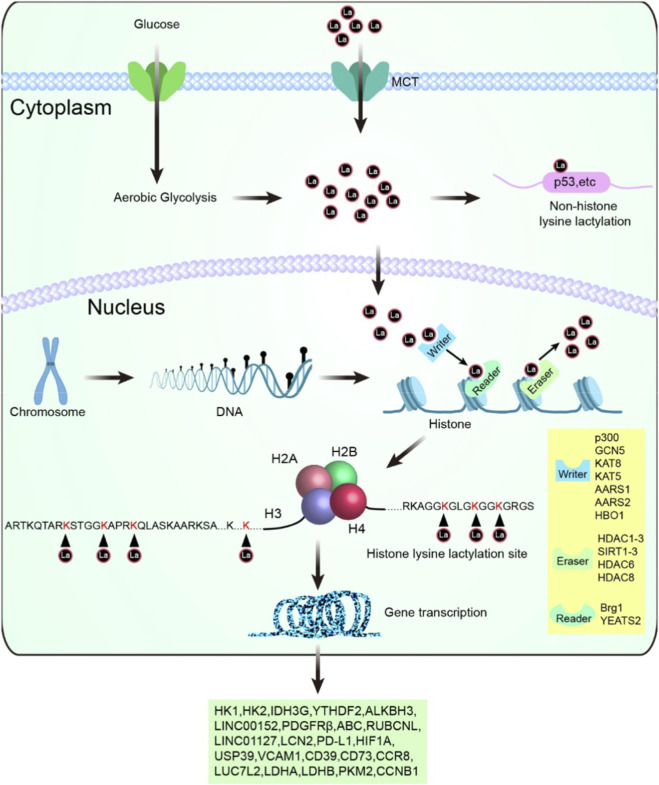
Lactylation Mechanisms: Histone and Non-Histone Modifications Driving Gene Regulation. Overview of lactylation processes and histone lysine lactylation. The schematic depicts the cellular processes involved in lactylation, originating from aerobic glycolysis and lactate production. Lactate, exported through monocarboxylate transporters (MCTs), enters the nucleus to modify histone and non-histone proteins through lysine lactylation. Histone lysine lactylation, mediated by writers (e.g., p300, KAT5, GCN5), erasers (e.g., HDAC1-3, SIRT1-3), and readers (e.g., Brg1, YEATS2), occurs at specific lysine residues such as H3K18 and H4K12, influencing chromatin structure and transcriptional activation.

These findings demonstrate that lactylation is neither an incidental nor an irreversible modification but rather a dynamic process under stringent enzymatic control. The establishment of its framework (Figure 3) endows lactylation with regulatory potential comparable to that of classical epigenetic modifications such as acetylation and methylation, thereby laying a solid theoretical foundation for in-depth exploration of p300-mediated histone and non-histone lactylation. In the following sections, we will further focus on the specific roles of p300 in lactylation regulation and systematically elucidate how p300 reshapes chromatin states and protein functions within lactate-rich tumor microenvironments to drive malignant progression.

## 4 p300-mediated lactylation: a metabolism-driven epigenetic execution layer

### 4.1 Histone lactylation: a p300-Orchestrated metabolism–chromatin positive feedback circuit

Lactylation and acetylation are considered two closely related lysine modifications that link cellular metabolic states to epigenetic regulation, exhibiting substantial overlap in substrate selection, spatial distribution, and functional output (Tian and Zhou, 2022). In this context, p300, a dual-functional catalytic core with both acetyltransferase and lactyl-transferase activities, undergoes a fundamental shift in its operational mode under lactate-rich conditions. Specifically, p300 transitions from a transcriptional co-activator primarily driven by acetylation to a metabolism-sensing epigenetic executor dominated by lactylation.

Early studies had already hinted at a potential role for p300 in metabolic regulation. In 2018, the Zhao’s group reported that loss of p300 markedly reduced the activity of key glycolytic enzymes, including ENO1, thereby impairing glycolytic capacity and rendering cells highly sensitive to glucose deprivation (Huang et al., 2018). This study first identified p300 as a lysine 2-hydroxyisobutyryltransferase involved in the regulation of glycolytic flux, providing early evidence for its central role in subsequent lactylation-dependent regulatory mechanisms. Subsequently, the same team further demonstrated that under Warburg effect–driven high-glycolytic conditions, p300 can catalyze histone lactylation by utilizing lactyl-coenzyme A through its HAT domain, solidifying its identity as a *bona fide* lactyl-transferase (Zhang et al., 2019). This discovery indicates that p300 does not merely respond passively to metabolic changes but actively converts metabolic intermediates into stable chromatin modifications, thereby directly reshaping transcriptional programs.

At the molecular level, p300-mediated histone lactylation predominantly occurs at transcriptionally active regions, with representative modification sites including lysine 18 on histone H3 (H3K18la) (Hu et al., 2025). These lactylation marks are frequently enriched at promoter or enhancer regions and show a strong positive correlation with transcriptional activation. Similar to acetylation, the formation of H3K18la weakens histone-DNA interactions, enhances chromatin accessibility, and facilitates the assembly of transcription factors and the transcriptional machinery. Critically, rather than acting uniformly across all malignancies, p300-mediated histone lactylation drives distinct oncogenic modules depending on the specific metabolic dependencies of the tumor microenvironment.

In highly glycolytic tumors, p300-mediated histone lactylation does not occur in isolation but is deeply embedded within a positive feedback network established by tumor metabolic reprogramming. In hepatocellular carcinoma models, p300-mediated acetylation of PDHX suppresses assembly of the pyruvate dehydrogenase complex (PDC), thereby blocking the entry of pyruvate into mitochondrial oxidative phosphorylation and leading to sustained lactate accumulation. Elevated lactate levels, in turn, drive p300-catalyzed histone lactylation events such as H3K56la, reshaping gene expression programs and ultimately promoting hepatocarcinogenesis (Jiang et al. (2024)). This process exemplifies a hierarchical regulatory loop encompassing acetylation-metabolic reprogramming-lactylation-transcriptional remodeling. Similar metabolism-epigenetic positive feedback circuits have been widely observed in other tumor types. In pancreatic cancer, H3K18la directly promotes transcriptional activation of acetyl-CoA acetyltransferase 2 (ACAT2), which subsequently induces acetylation of mitochondrial carrier homolog 2 (MTCH2). This cascade stabilizes MTCH2 while disrupting oxidative phosphorylation, further enhancing glycolytic flux and exacerbating lactate production, thereby establishing a self-reinforcing loop that sustains high lactate levels in tumor cells (Yang J. et al., 2025).

Beyond intrinsic metabolic reinforcement, H3K18la critically governs classical oncogenic signaling and metastatic cascades across diverse tumor types. In non-small cell lung cancer (NSCLC), enrichment of H3K18la at the YTHDF2 promoter markedly elevates its transcription. In-creased YTHDF2 expression facilitates m6A-dependent degradation of SFRP2 mRNA, thereby relieving inhibition of the Wnt signaling pathway and driving malignant progression (Zhao S. et al., 2025). Concurrently, topoisomerase TOP2A enhances p300 expression and promotes LDHA-mediated lactate production, further elevating H3K18la levels and synergistically accelerating NSCLC development and progression (Wu Q. et al., 2025). Crucially, many of these downstream oncogenic cascades, such as the H3K18la-YTHDF2 axis could be pharmacologically abrogated by the specific p300 catalytic inhibitor A-485, providing direct functional evidence that p300-mediated lactylation is a requisite driver of these transcriptional programs. Similarly, in the context of tumor dissemination, in colorectal cancer, p300 forms a complex with the chromatin remodeling factor BRG1 and modulates H3K18la to alter chromatin accessibility, thereby enriching the transcription fac-tor ERG and activating MMP9 expression, ultimately promoting liver metastasis (Lv et al., 2025). Remarkably, in these models, p300 functions not only as a catalytic driver of lactylation but also as a signal amplification node, whereby its catalytic products feedback to enhance lactate production or p300 expression itself, forming self-sustaining malignant circuits. This p300-centered lactylation feedback mechanism is particularly evident in pancreatic ductal adenocarcinoma (PDAC). Lactate accumulation drives H3K18la enrichment at the promoters of TTK and BUB1B, activating their transcription. TTK subsequently phosphorylates and activates LDHA to enhance lactate pro-duction, while TTK and BUB1B positively regulate p300 expression, collectively establishing a malignant network in which metabolic reprogramming, epigenetic regulation, and cell cycle dysregulation are tightly coupled (Li et al., 2024). Similar mechanisms have been validated in NSCLC and hepatocellular carcinoma, where the NNMT-ALDH3A1 axis and YBX1-mediated positive feedback pathways, respectively, amplify H3K18la signaling (Dai J. et al., 2025; Ji et al., 2025).

It is worth noting that p300-mediated histone lactylation is not a unidirectional or uncontrolled process but is dynamically regulated by upstream signals. In non-tumor contexts such as viral infection, host cell lactate accumulation can similarly drive p300-mediated H3K18la and H4K12la modifications, promoting expression of viral replication–associated genes. Meanwhile, viruses themselves can encode miR-N20 to suppress HIF-1α, thereby limiting glycolysis and lactate production at the source to avoid detrimental consequences of excessive lactylation (Zhang and Zhang, 2024). These observations suggest that p300-mediated lactylation represents not only an oncogenic mechanism but also a highly tunable adaptive response.

In summary, p300-mediated histone lactylation serves as more than a mere execution layer; it functions as a critical metabolic-to-chromatin transducer. By converting the transient accumulation of lactate into durable epigenetic memory, p300 solidifies the Warburg effect into a stable transcriptional program. This process does not simply sustain malignant phenotypes but effectively locks tumor cells into an aggressive state. Consequently, targeting p300 offers a precision therapeutic opportunity to dismantle this metabolic-epigenetic addiction, going beyond general metabolic intervention to strike at the root of tumor plasticity.

### 4.2 Non-histone lactylation: immediate protein functional reprogramming bypassing transcription

Following the establishment of p300 as a histone lactyl-transferase that drives transcriptional reprogramming through chromatin remodeling, a more critical question naturally arises: must lactate signals necessarily act through the transcriptional layer to influence cell fate? Recent studies provide a clear negative answer. p300-mediated non-histone lactylation enables lactate to bypass transcription and chromatin remodeling, directly and rapidly imposing instant reprogramming on functional proteins (Shegay et al., 2022). In doing so, it markedly expands the temporal resolution and regulatory precision through which metabolic signals exert their biological effects.

Similar to non-histone acetylation, p300-mediated non-histone lactylation displays pronounced substrate selectivity. This selectivity is not dictated by chromatin localization but instead relies on direct interactions between p300 and specific protein substrates, allowing lactylation to be precisely installed at functionally critical lysine residues. Through this mechanism, lactate is transformed from a metabolic byproduct into a molecular instruction capable of precisely modulating protein conformation, interaction networks, and subcellular localization.

At the level of post-transcriptional regulation, intrahepatic cholangiocarcinoma (iCCA) provides a representative model illustrating the functional impact of non-histone lactylation. Enhanced glycolysis in tumor cells drives p300-mediated lactylation of the nucleolar protein nucleolin (NCL) at K477. This modification reshapes mRNA splicing programs to upregulate MAP kinase-activating death domain protein (MADD), thereby activating ERK signaling and continuously promoting tumor progression (Yang et al., 2024a). This example clearly demonstrates the capacity of lactylation to directly rewire signaling pathways without altering chromatin states. Beyond RNA processing, and highlighting its functional versatility, p300-mediated non-histone lactylation can profoundly influence tumorigenesis by regulating RNA stability. In renal cell carcinoma (RCC), p300 lactylates the m6A reader protein YTHDC1 at K82, promoting the formation of highly stable nuclear condensates. These YTHDC1 condensates selectively sequester multiple oncogenic mRNAs, including transcripts encoding BCL2 and E2F2, thereby preventing their recognition and degradation by the PAXT-exosome complex. The resulting enhancement of anti-apoptotic and pro-proliferative protein expression confers a pronounced survival advantage to RCC cells (Dai C. et al., 2025). In contrast to its role in RNA dynamics, p300 also specifically targets components of the metabolic machinery to enforce a self-sustaining Warburg effect. For example, in bladder cancer, a lactate-rich environment drives p300-mediated lactylation of the RNA-binding protein HNRNPA1 at K350, biasing alternative splicing toward the generation of the glycolysis-promoting PKM2 isoform. Increased PKM2 expression further enhances glycolytic flux and lactate production, thereby establishing a positive feedback loop that sustains malignant metabolic states (Wang X. et al., 2025). This model illustrates that non-histone lactylation is not merely a passive response to metabolic alterations but an active driver of metabolic reprogramming.

Significantly, p300-mediated non-histone lactylation is not confined to nuclear proteins. Its direct regulation of organelle-associated functions further extends the spatial dimension of lactylation signaling. While the aforementioned targets operate primarily within the nucleocytoplasmic space, p300 also critically modulates vesicular trafficking. In hepatocellular carcinoma, p300 lactylates Rab7A, a GTPase that regulates late endosome and multivesicular body trafficking, thereby suppressing its GTPase activity. This inhibition promotes sustained trafficking of multivesicular bodies to the plasma membrane and excessive release of tumor-derived exosomes. These exosomes are enriched in integrin β4 and extracellular matrix remodeling proteins, creating a permissive niche for distant metastasis of hepatocellular carcinoma, particularly lung metastasis (Jiang et al., 2025). Even more strikingly, and representing a dramatic functional repurposing, p300-mediated non-histone lactylation can endow classical immune checkpoint molecules with entirely new, non-immunological functions. In hepatocellular carcinoma models, p300 catalyzes lactylation of programmed death-ligand 1 (PD-L1) at K189. As validated by genetic K189R site-directed mutagenesis, this specific modification, rather than enhancing its canonical immunosuppressive activity, this modification promotes insulin-dependent nuclear translocation of PD-L1. Within the nucleus, lactylated PD-L1 directly activates transcriptional programs governing cholesterol bio-synthesis, thereby markedly accelerating tumor growth (Wang X. et al., 2025). This finding highlights the remarkable potential of lactylation to functionally redefine protein identities.

Taken together, p300-mediated non-histone lactylation constitutes a highly flexible and rapidly responsive regulatory system that enables tumor cells to instantaneously convert lactate-rich metabolic states into multilayered functional outputs. By simultaneously targeting RNA processing, protein homeostasis, metabolic pathways, organelle trafficking, and immune checkpoint functions, this system directly drives the establishment and maintenance of malignant phenotypes without relying on transcriptional remodeling. Of note, the biological effects described across these diverse models are increasingly supported by direct functional studies rather than mere correlative observations. While early evidence often relied on the co-occurrence of elevated intratumoral lactate and global lactylation enrichment, the critical oncogenic pathways discussed here have been rigorously established as causal mechanisms. These include PD-L1 nuclear translocation and YTHDC1 condensate formation. Utilizing stringent genetic manipulations including targeted p300 ablation via shRNA or CRISPR and site-directed mutagenesis such as K-to-R substitutions at key functional lysines (Wang X. et al., 2025; Dai C. et al., 2025), researchers have effectively uncoupled lactylation from metabolic correlation, confirming its autonomous role as a driver of tumor plasticity.

The recognition of this regulatory layer not only further consolidates p300 as a central metabolic-epigenetic hub but also provides a new conceptual dimension for understanding tumor metabolic adaptability and for developing precision therapeutic interventions. In order to provide a systematic overview of this landscape, Table 2 summarizes representative histone and non-histone modification sites, their specific molecular mechanisms (with an emphasis on functionally validated pathways), and relevant cancer models.

**TABLE 2:** Functional Landscape of p300-Mediated Lactylation in Cancer. This table summarizes representative histone and non-histone lactylation events catalyzed by p300 in cancer. Listed are the specific lactylation sites, their protein substrates (histone or non-histone), the primary oncogenic functions or pathways affected, and the associated cancer types.

Substrate (histone/Non-histone)	Lactylation site	Function	Cancer type	Ref.
H3	K56la	Activates oncogenic transcription	HCC	Jiang et al. (2024)
H3	K18la	Amplifies lactate production	PACA	Yang J. et al. (2025)
		Derepresses Wnt signaling pathway	NSCLC	Zhao et al. (2025b)
		Promotes liver metastasis	CRC	Lv et al. (2025)
		Exacerbates lactate production, Disrupts cell cycle regulation	PDAC	Li et al. (2024)
		Exacerbates lactate production	NSCLC	Dai C. et al. (2025)
		Exacerbates lactate production	HCC	Ji et al. (2025)
NCL	K477la	Activates ERK signaling pathway	iCCA	Yang et al. (2024a)
YTHDC1	K82la	Confers survival advantage	RCC	Dai C. et al. (2025)
HNRNPA1	K350la	Enhances glycolytic flux	Bladder Cancer	Wang T. et al. (2025)
Rab7A	—	Mediates exosome hypersecretion	HCC	Jiang et al. (2025)
PD-L1	K189la	Promotes cholesterol biosynthesis	HCC	Wang X. et al. (2025)

### 4.3 Upstream regulation of p300: post-translational modifications as a catalytic switch

Having established the extensive landscape of p300-mediated acetylation and lactylation, a critical scientific question emerges: how is p300 itself regulated to execute these specific metabolic-epigenetic functions? The catalytic activity, stability, and substrate preference of p300 are tightly governed by a complex network of upstream post-translational modifications (PTMs) that integrate extracellular signals with nuclear responses.

Phosphorylation represents the most dynamic layer of p300 regulation, often acting as a molecular switch for its enzymatic activity. In the study of hepatic fibrosis, it has been demonstrated that increased matrix stiffness activates RHOA-dependent AKT signaling in hepatic stellate cells (HSCs). This activation directly induces p300 phosphorylation at Ser1834, facilitating its nuclear translocation and subsequent transcription of pro-fibrotic genes (Dou et al., 2018). Similarly, in the context of gallbladder cancer (GBC), another research group reported that a stiff microenvironment promotes cancer cell malignancy through the same PTM of p300, albeit via a distinct upstream mechanism involving stromal cells (Sun et al., 2025). Furthermore, emerging evidence suggests a reciprocal regulatory loop between the transcriptional coactivator p300 and the PI3K-AKT signaling axis, wherein p300 appears to sustain AKT activation through a positive feedback mechanism. Specifically, p300 has been reported to upregulate CSNK2A1 expression via the PI3K-AKT-mTOR axis. The resulting CSNK2A1, in turn, further amplifies this signaling cascade, as evidenced by increased phosphorylation of AKT at both Ser473 and Thr308. This reciprocal activation establishes a self-amplifying loop: microenvironmental cues trigger p300 activation through AKT, while p300 subsequently reinforces AKT signaling via CSNK2A1-mediated feedback, collectively driving EMT and tumor progression (Liu, 2023).

Accumulating evidence establishes p300 as a central hub that integrates diverse upstream signals through site-specific phosphorylation. Beyond the mechanical signaling axis discussed above, p300 is also subject to regulation by inflammatory and stress-associated kinases. For instance, a recent study investigating cancer-associated cachexia revealed a distinct regulatory mechanism. The authors demonstrated that cancer-induced activation of Toll-like receptor 4 in skeletal muscle triggers p38β MAPK signaling, which directly phosphorylates p300 at a previously unidentified site, serine 12 (Sin et al., 2020). This phosphorylation enhances p300's acetyltransferase activity toward C/EBPβ, leading to the upregulation of catabolic genes and subsequent muscle wasting. Notably, this modification was shown to be both necessary and sufficient for the cachectic phenotype. Collectively, these findings illustrate that p300 activity is dynamically regulated by site-specific phosphorylation. As a result, p300 contributes to distinct cancer-related phenotypes depending on which phosphorylation event occurs.

In contrast to the positive regulation of p300 by AKT signaling discussed above, a distinct regulatory paradigm has emerged from studies on Parkinson’s disease. In this context, AMPK exerts negative control over p300. A recent study demonstrated that pathogenic α-Synuclein mutations activate ACLY. This leads to acetyl-CoA accumulation and subsequent AMPK inhibition. The suppression of AMPK dramatically alters p300 subcellular localization (Son et al., 2025). Specifically, it reduces p300 availability in the nucleus while increasing its retention in the cytoplasm. This compartment-specific dysregulation has dual consequences. It impairs epigenetic signaling through reduced histone acetylation. It also disrupts autophagic flux via Raptor hyper-acetylation and subsequent mTORC1 activation. Collectively, these events contribute to neurodegeneration.

Moreover, p300 relies on autoacetylation within its catalytic loop to relieve structural auto-inhibition and achieve full activation. Given its promiscuous acyltransferase nature, whether an analogous auto-lactylation occurs under high-lactate conditions to conformationally lock p300 into a lactyl-transferase state remains a fascinating, yet unverified, hypothesis for future investigation.

### 4.4 p300-mediated lactylation in tumor microenvironment remodeling and therapeutic resistance

Far from being a monolithic aggregation of malignant clones, the neoplastic lesion represents a sophisticated ecosystem comprising diverse cell lineages, including fibroblasts, vascular endothelial cells, and immune infiltrates embedded within a dynamic extracellular matrix (Sun et al., 2024). The intricate crosstalk between metabolic fluxes and inflammatory signaling within this milieu dictates disease progression and treatment outcomes (Wang Z. et al., 2025). Within this complex ecosystem, p300 emerges as a decisive metabolic integrator. By translating the lactate-rich environment into stable epigenetic and proteomic signatures, p300-mediated lactylation effectively locks the cellular machinery into a pro-tumorigenic state, thereby driving stromal reprogramming and therapeutic recalcitrance.

#### 4.4.1 p300-dependent lactylation in tumor angiogenesis and vascular abnormalities

The mechanistic execution of p300-driven angiogenesis relies on its ability to directly couple metabolic signals to specific transcriptional programs. Beyond general co-activation, p300 utilizes lactyl-CoA to catalyze the specific lactylation of the transcription factor YY1, predominantly at the K183 residue. This modification serves as a critical molecular switch, significantly enhancing the DNA-binding affinity and transcriptional output of YY1 towards key angiogenic effectors like FGF2. Consequently, this p300-YY1-FGF2 axis acts as a potent driver of neovascularization in high-lactate tumor cores. Crucially, pharmacological blockade of this axis using the p300-selective inhibitor A485 effectively severs this metabolic-transcriptional link, suppressing aberrant angiogenesis in both *in vitro* and *in vivo* models (Peng et al., 2025; Zhu et al., 2025; Wang X. et al., 2023). These findings directly establish the role of p300-dependent lactylation in pathological tumor vascularization.

Parallel to activating angiogenic factors, p300-mediated signaling fundamentally rewires endothelial identity through the Endothelial-to-Mesenchymal Transition (EndoMT). This process is governed by the epigenetic modulation of master regulators such as Snail1. Under metabolic stress, p300-mediated modification of Snail1 amplifies the TGF-β/Smad signaling cascade, thereby stripping endothelial cells of their barrier function and conferring a migratory, invasive phenotype (Fan et al., 2023). While primarily characterized in oncology, the universality of this vascular plasticity mechanism is corroborated by findings in vascular inflammatory diseases. For instance, in atherosclerosis models, which share chronic inflammatory features with the TME, the histone chaperone ASF1A cooperates with p300 to sustain H3K18la levels and SNAI1 transcription; its deletion effectively halts EndoMT (Dong et al., 2024). Furthermore, p300-dependent chromatin remodeling has been linked to the establishment of long-term epigenetic memory in response to metabolic insults, suggesting that p300 may imprint a lasting vascular memory that predisposes vessels to dysfunction (Li et al., 2025a). Collectively, these data delineate a unified model wherein p300 integrates metabolic cues to drive both physiological angiogenesis and pathological vascular remodeling.

#### 4.4.2 p300-dependent lactylation in immune microenvironment regulation and immune evasion

A lactate-rich microenvironment is widely recognized as a major suppressor of antitumor immunity. Within this context, p300-dependent lactylation functions as a dual-edged immunomodulator: it reprograms tumor-intrinsic pathways to evade recognition while simultaneously impairing immune effector functions. In macrophages, p300 serves as a bridge between metabolism and cell fate. Recent evidence reveals that p300-dependent H3K18la upregulates METTL3, which in turn accelerates SLC7A11 mRNA degradation via the m6A-YTHDF2 axis. This cascade relieves the inhibition of ferroptosis, driving macrophages towards ferroptotic death and exacerbating inflammation—a mechanism likely conserved between atherosclerotic plaques and the inflamed tumor stroma (Chen et al., 2025).

At the tumor cell level, p300 further fortifies the immunosuppressive barrier. p300-mediated lactylation of PD-L1 not only confers novel nuclear functions that promote tumorigenesis but may also compromise the efficacy of immune checkpoint blockade through metabolic interference (Wang X. et al., 2025). Additionally, the synergistic deposition of H3K27ac and H3K18la by p300 has been shown to amplify PD-L1 transcription, thereby establishing a robust immunosuppressive microenvironment across multiple malignancies (Wang et al., 2024).

#### 4.4.3 p300-mediated lactylation and therapeutic resistance: adaptive evolution driven by metabolic stress

Therapeutic resistance remains one of the principal obstacles limiting durable clinical responses in cancer treatment. Accumulating evidence indicates that p300-mediated lactylation confers strong adaptive advantages to tumor cells by converting metabolic stress into stable epigenetic and protein functional alterations. In resistance mechanisms associated with the DNA damage response, lactylation can attenuate therapeutic efficacy by enhancing DNA repair programs.

Firstly, p300 fortifies the DNA damage response (DDR). In glioblastoma, p300-driven H3K9la promotes resistance to temozolomide by upregulating repair genes like LUC7L2 while suppressing the mismatch repair gene MLH1 (Yue et al., 2024). Similarly, H4K12la, regulated by AKR1B10, enhances DNA replication dynamics to mediate resistance to pemetrexed (Duan et al., 2023). Secondly, p300 orchestrates metabolic plasticity and cytoprotection. In colorectal cancer, H3K18la drives RUBCNL expression to induce bevacizumab resistance. In glioma, p300 interacts with succinyl-CoA synthetase to utilize local lactyl-CoA pools, upregulating GDF15 to promote radioresistance (Liu et al., 2024). Furthermore, p300 activates protective autophagy to negate therapeutic stress. For instance, in pancreatic cancer, H3K18la induces HNRNPC expression, which stabilizes TRAF6 mRNA to trigger autophagy, thereby clearing chemotherapy-induced damage (Huang et al., 2025). Concurrently, this axis stabilizes ALDH1A3 to enhance glycolysis, fueling the energy demands of this resistance phenotype. Notably, these mechanisms often culminate in metabolism-epigenetic positive feedback loops. For example, in colon cancer stem cells, LDHA-driven lactate induces p300-dependent H4K12la, which in turn upregulates GLHC to suppress ferroptosis and confer oxaliplatin resistance (Deng et al., 2025).

Overall, p300-mediated lactylation orchestrates a multi-layered remodeling of both the TME and intrinsic cellular defense networks. This framework explains why lactate-rich tumors exhibit such aggressive evolutionary plasticity and provides a compelling rationale for targeting the p300-metabolism axis to dismantle therapeutic resistance.

## 5 Discussion and perspectives: p300 as a metabolic-epigenetic integrative Hub—Theoretical significance and therapeutic implications

This review systematically summarizes the multi-layered roles of p300-mediated acetylation and lactylation in tumor initiation, progression, and therapeutic resistance. Collectively, current evidence indicates that p300 is not a single-function acetyltransferase in the traditional sense, but rather a dynamic metabolic-epigenetic integrative hub capable of sensing cellular metabolic states and switching its catalytic preferences among distinct post-translational modifications. This dual-functional property enables p300 to convert transient and reversible metabolic signals into relatively stable epigenetic and protein functional alterations, thereby profoundly shaping tumor biology.

### 5.1 Conceptual integration of the dual functions of p300: from transcriptional Co-activator to metabolism-sensing executor

Conventionally, p300 has been defined as a classical transcriptional coactivator. Its core function involves opening chromatin structure through histone acetylation to enhance gene transcription (Picavet et al., 2024). However, recent advances in metabolic epigenetics have fundamentally expanded this conceptual framework. Mechanistically, p300 functions as a highly promiscuous acyltransferase. It can utilize multiple acyl donors, including acetyl-CoA, lactyl-CoA, crotonyl-CoA, and succinyl-CoA. Recent evidence highlights this enzymatic versatility by identifying p300 as a direct writer of histone crotonylation. For example, the p300 enzyme specifically deposits crotonyl groups at the H3K18 locus across active promoters to drive gene transcription and ensure successful early embryonic development (Gao et al., 2024). Furthermore, this catalytic adaptability extends to lysine succinylation in malignant contexts. In lung adenocarcinoma, cytoplasmic p300 directly succinylates key glycolytic enzymes such as PGK1. This specific non-histone modification accelerates glucose metabolic reprogramming and significantly enhances cellular lactic acid excretion (Ma et al., 2024). Because p300 actively processes these diverse metabolic intermediates, its broad substrate affinity creates an intense competitive landscape among various acylation modifications within the cell.

This dynamic competition is primarily governed by the stoichiometry of intracellular acyl-CoA species. Under metabolically homeostatic conditions, the acetyltransferase activity of p300 predominates, maintaining normal transcriptional programs via canonical modifications such as H3K18ac and H3K27ac (Zaghi et al., 2023). In contrast, the Warburg effect generates a highly lactate-rich tumor microenvironment. The surge in intracellular lactate drives lactyl-CoA synthesis, enabling this metabolite to outcompete acetyl-CoA and other short-chain acyl-CoAs for the p300 catalytic pocket. Direct substrate competition is particularly evident at specific histone lysine residues. For example, H3K18 can be either acetylated or lactylated depending on the dominant metabolic flux. The epigenetic switch from H3K18ac to H3K18la often signifies a critical cellular transition. It marks the shift from homeostatic gene expression to a stress-responsive oncogenic program. Importantly, this catalytic shift is unlikely to result from substrate competition alone. It likely reflects the integrated effects of p300 conformational changes, regulatory protein interactions, and specific chromatin contexts. Through these mechanisms, p300 dynamically reallocates its catalytic capacity based on the availability of metabolic intermediates. This dual-functional model provides a unified theoretical framework for understanding the coupling between tumor metabolism and epigenetic regulation. Tumor cells do not passively endure the consequences of metabolic alterations. Instead, they utilize nodal regulators like p300 to actively translate metabolic stress into genetic programs that ensure survival and evolutionary fitness.

### 5.2 Site specificity and gene selectivity: unresolved questions in p300-Mediated lactylation

While the substrate competition between acetyl-CoA and lactyl-CoA beautifully explains how p300 senses the metabolic state, it raises a critical unresolved question: how does p300 achieve site specificity? Despite extensive reports of p300-mediated lactylation across multiple tumor types, the principles governing site specificity and gene selectivity remain among the most critical unresolved issues in the field. Available evidence suggests that p300-dependent lactylation does not occur randomly across the genome but is highly enriched at specific promoters, enhancers, or functionally critical lysine residues on target proteins (Henry et al., 2013). Such selectivity is likely dictated by multiple, coordinated mechanisms.

The specificity of p300-mediated lactylation is governed by a sophisticated interplay between metabolic channeling and genomic architecture, rather than being a stochastic enzymatic event. Central to this regulation is the spatial coupling of metabolism and epigenetics: key glycolytic enzymes, such as LDHA and PKM2, physically associate with p300 to generate localized pools of lactyl-coenzyme A. This proximity-driven mechanism ensures a high-concentration substrate supply directly at target chromatin regions (Wu Q. et al., 2025; Wang T. et al. (2025); Liu Z. et al., 2025). Concurrently, the genomic specificity of these modifications is dictated by the recruitment of p300 via sequence-specific transcription factors and chromatin remodeling complexes, which direct the enzyme to precise loci (Liu et al., 2016). Furthermore, this process operates within a hierarchical epigenetic framework where pre-existing marks, particularly H3K27ac, function as molecular anchors. These acetylation signals prime specific regions for subsequent lactylation, thereby facilitating a coordinated crosstalk between distinct acyl modifications within the same regulatory landscape.

In addition, the post-translational modification status of p300 itself (including phosphorylation and acetylation), as well as its interactions with auxiliary cofactors, may fine-tune its lactylation output by modulating conformational stability and substrate affinity (Masaki et al., 2023). Future integration of multi-omics approaches including CUT&Tag, ChIP-seq, spatial transcriptomics, and metabolomics will be instrumental in con-structing high-resolution regulatory maps of p300-mediated lactylation (Liu J. et al., 2025).

### 5.3 Therapeutic implications: precision targeting of the dual functional activities of p300

From a therapeutic perspective, the dual-functional nature of p300 presents both opportunities and challenges. On one hand, the central roles of p300-mediated acetylation and lactylation in tumor progression and resistance make it an attractive therapeutic target. On the other hand, the essential functions of p300 in normal physiology and immune activation raise concerns that global inhibition strategies may incur substantial adverse effects.

Accordingly, future therapeutic strategies may need to shift from “pan-inhibition” toward “function-selective intervention.” For example, in highly glycolytic, lactate-rich tumors, preferential inhibition of the lactyl-transferase activity of p300 or disruption of localized lactyl-coenzyme A generation may attenuate tumor adaptability while preserving the physiological roles of acetylation. Conversely, in specific con-texts, stabilizing or enhancing p300-mediated acetylation to activate tumor suppressor genes or immune-related pathways may confer therapeutic benefit (Zamperla et al., 2024).

To overcome therapeutic resistance, future strategies must move beyond monotherapy, focusing instead on co-targeting p300 alongside critical vulnerabilities such as metabolic checkpoints, DNA repair machinery, immune evasion pathways, or ferroptosis regulators. This combinatorial logic aims to collapse the metabolism–epigenetic circuitry that sustains tumor survival (Mokhtar, 2025; Wali et al., 2025). Capitalizing on recent technological breakthroughs, including computational drug discovery, PROTAC-based degradation, and spatial metabolomics, researchers are now poised to engineer next-generation p300 modulators with unprecedented selectivity and potency (Vannam et al., 2021; Fiorentino et al., 2025).

## 6 Concluding remarks

**FIGURE 4:**
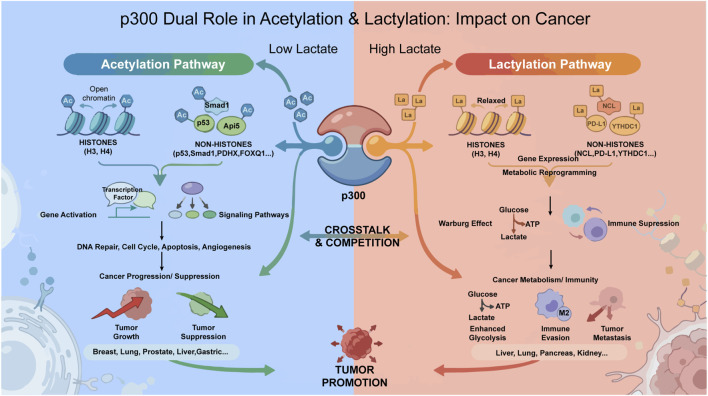
Dual roles of p300-mediated acetylation and lactylation and their impact on cancer. The schematic illustrating how lactate availability biases p300 toward distinct post-translational modifications with divergent consequences for cancer. Under low lactate (left), p300 primarily acetylates histones H3 and H4 and non-histone substrates (p53, Smad1, PDHX, FOXQ1, etc.), resulting in chromatin relaxation and transcriptional activation. These changes interface with transcription factors and signaling pathways to influence DNA repair, cell-cycle control, apoptosis, and angiogenesis, yielding context-dependent effects on tumor progression or suppression. Under high lactate (right), p300 activity favors lysine lactylation of histones H3 and H4 and non-histone proteins (NCL, PD-L1, YTHDC1, etc.), driving metabolic reprogramming and a Warburg-like phenotype and contributing to immune suppression and immune evasion, thereby promoting tumor growth and metastasis. This figure highlights crosstalk and competition between acetylation and lactylation downstream of p300. The processes depicted are implicated across multiple malignancies, including breast, lung, prostate, liver, gastric, pancreatic, and renal cancers.

In conclusion, the dual enzymatic identity of p300 governing both acetylation and lactylation constitutes a highly dynamic and context-dependent regulatory apparatus that transcends classical chromatin biology. Far from being a passive writer, p300 functions as a sophisticated metabolic sensor and epigenetic transducer. It enables tumor cells to seamlessly translate transient metabolic fluctuations into durable and heritable transcriptional programs. This plasticity allows malignancies to navigate hostile microenvironments, evade immune surveillance, and acquire therapeutic resistance, effectively converting metabolic stress into a survival advantage (Figure 4).

A pivotal insight emerging from this review is the competitive yet synergistic relationship between acetyl-CoA and lactyl-CoA within the p300 catalytic pocket. This biochemical duality implies that the cellular epigenetic landscape is not static but is continuously reshaped by local metabolite availability. Consequently, p300-mediated lactylation represents a distinct layer of regulation that specifically locks in a pro-tumorigenic state under hypoxic and glycolytic conditions, distinguishing it functionally from canonical acetylation.

Looking forward, the field stands on the precipice of major technological breakthroughs required to resolve the remaining ambiguities in p300-mediated lactylation. Future research must prioritize three specific technical directions to fully decode this epigenetic signaling layer.

First, advanced structural elucidation utilizing high-resolution cryo-electron microscopy will be essential. While early structural studies successfully resolved the p300 catalytic core bound to conventional acetyl-CoA (Ortega et al., 2018; Yu et al., 2023), capturing the intact ternary complex of p300, lactyl-CoA, and the nucleosome remains a critical unresolved challenge (Herbst et al., 2021; Zhang et al., 2022). This fundamental insight is necessary to define the precise structural basis of substrate preference and will directly facilitate the rational design of lactylation-specific inhibitors that spare essential acetylation functions. Second, innovating chemical biology tools is critical for tracking lactylation dynamics in live cells. Researchers must develop site-specific anti-lactyllysine antibodies to replace current broad-spectrum pan-antibodies. Furthermore, engineering advanced biosensors utilizing fluorescence resonance energy transfer will enable the real-time observation of lactylation events with unprecedented spatiotemporal resolution. Third, the implementation of single-cell multi-omics will revolutionize our understanding of tumor heterogeneity. Integrating single-cell CUT&Tag sequencing with spatial metabolomics will allow researchers to map the precise distribution of histone lactylation across different tumor cell subpopulations. Such high-resolution approaches will decipher how distinct metabolic niches drive localized epigenetic remodeling, allowing clinicians to distinguish between therapeutic-resistant clones and sensitive cell populations.

A deeper understanding of p300 as a metabolic–epigenetic integrative hub will not only elucidate fundamental principles of tumor biology but also provide critical theoretical support for the rational design of precision-targeted therapies. This theoretical foundation is especially crucial because, while aberrant p300-mediated lactylation is increasingly correlated with poor patient outcomes in clinical tumor cohorts, systemic pan-inhibition of p300 poses profound physiological challenges due to its indispensable baseline functions in normal development and immune homeostasis. Beyond broad-spectrum enzymatic inhibitors, the development of next-generation targeted protein degraders such as PROTACs offers unprecedented opportunities to selectively eliminate specific p300 functions (Durbin et al., 2021). By disrupting the feedback loops that sustain tumor metabolic addiction, these novel therapeutic strategies could sensitize refractory tumors to standard-of-care treatments including immunotherapy and DNA-damaging agents. Moreover, the conceptual framework elucidated here extends well beyond cancer. It offers a universal model for understanding other pathologies driven by metabolic dysregulation such as fibrosis, cardiovascular disease, and chronic inflammation. As research continues to unravel the complexities of this writer, p300 is poised to transition from a classic transcriptional co-activator to a central target in the next-generation of metabolism-based therapeutics.
